# Infectious sacroiliitis: a retrospective, multicentre study of 39 adults

**DOI:** 10.1186/1471-2334-12-305

**Published:** 2012-11-15

**Authors:** Marion Hermet, Emeline Minichiello, René Marc Flipo, Jean Jacques Dubost, Yannick Allanore, Jean Marc Ziza, Philippe Gaudin, Thierry Thomas, Emmanuelle Dernis, Baptiste Glace, Alain Regnier, Martin Soubrier

**Affiliations:** 1 Service de Médecine Interne, Hôpital G. Montpied, CHU de Clermont-Ferrand, 58 rue Montalembert, Cedex 1, Clermont-Ferrand 63003, France; 2 Service de Rhumatologie, Hôpital R. Salengro, CHU de Lille, Lille, France; 3 Service de Rhumatologie, Hôpital G. Montpied, CHU de Clermont-Ferrand, Clermont-Ferrand, France; 4 Service de Rhumatologie A, Université Paris Descartes, Hôpital Cochin, Paris, France; 5 Service de Rhumatologie, Hôpital de la Croix St Simon, Paris, France; 6 Service de Rhumatologie, Hôpital Sud, CHU de Grenoble, Grenoble, France; 7 Service de Rhumatologie, Hôpital Nord, CHU de St Etienne, St Etienne, France; 8 Service de Rhumatologie, Centre Hospitalier, Le Mans, France; 9 Service de Rhumatologie, Hôpital J. Lacarin, CH de Vichy, Vichy, France

## Abstract

**Background:**

Non-brucellar and non-tuberculous infectious sacroiliitis (ISI) is a rare disease, with misleading clinical signs that delay diagnosis. Most observations are based on isolated case reports or small case series. Our aim was to describe the clinical, bacteriological, and radiological characteristics of ISI, as well as the evolution of these arthritis cases under treatment.

**Methods:**

This retrospective study included all ISI cases diagnosed between 1995 and 2011 in eight French rheumatology departments. ISI was diagnosed if sacroiliitis was confirmed bacteriologically or, in the absence of pathogenic agents, if clinical, biological, and radiological data was compatible with this diagnosis and evolution was favourable under antibiotic therapy.

**Results:**

Overall, 39 cases of ISI were identified in adults, comprising 23 women and 16 men, with a mean age at diagnosis of 39.7 ± 18.1 years. The left sacroiliac joint (SI) was affected in 59% of cases, with five cases occurring during the post-partum period. Lumbogluteal pain was the most common symptom (36/39). Manipulations of the SI joint were performed in seven patients and were always painful. Mean score for pain using the visual analogue score was 7.3/10 at admission, while 16 patients were febrile at diagnosis. No risk factor was found for 30.7% of patients. A diagnosis of ISI was only suspected in five cases at admission. The mean time to diagnosis was long, being 43.3 ± 69.1 days on average. Mean C-reactive protein was 149.7 ± 115.3 mg/l, and leukocytosis (leukocytes ≥ 10 G/l) was uncommon (n = 15) (mean level of leukocytes 10.4 ± 3.5 G/l). Radiographs (n = 33) were abnormal in 20 cases, revealing lesions of SI, while an abdominopelvic computed tomography (CT) scan (n = 27) was abnormal in 21 cases, suggesting arthritis of the SI joints in 13 cases (48.1%) and a psoas abscess in eight. Bone scans (n = 14) showed hyperfixation of the SI in 13 cases. Magnetic resonance imaging (MRI) (n = 27), when focused on the SI (n = 25), directed towards the diagnosis to ISI in 25 cases. Pathogenic agents were isolated in 33 cases (84.6%) by means of articular puncture (n = 16), blood culture (n = 14), cytobacteriological examination of urine (n = 2), or puncture of the psoas (n = 1).

Gram-positive cocci were the mostly isolated common bacteria, with a predominance of staphylococci (n = 21). The most frequently isolated gram-negative bacillus was *Pseudomonas aeruginosa* (n = 3). Evolution was favourable in 37 out of 39 patients under prolonged antibiotic therapy (mean duration 3.01 ± 1.21 months).

**Conclusion:**

Our series confirmed that the clinical manifestations of ISI usually lead to delayed diagnosis. Based on our results, we suggest performing an MRI of the spine and SI in clinical situations characterised by lumbogluteal pain and symptoms of an infectious disease, such as fever.

## Background

Non-brucellar and non-tuberculous infectious sacroiliitis (ISI) is a rare disease, involving between 1 and 2% of septic arthritis cases [1], with misleading signs that delay diagnosis. Most observations are based on single case reports or small case series. This study was aimed to review the clinical, bacteriological, and radiological characteristics of ISI, along with the evolution of this type of arthritis under treatment.

## Methods

The study included all patients with ISI hospitalised between 1995 and 2011 in the rheumatology departments of six university hospitals and two general hospitals in France. ISI was diagnosed if there was bacteriological proof of infection or, in the absence of pathogenic agents, if the clinical, biological, and radiological data was compatible with this diagnosis and evolution was favourable under antibiotic therapy. Cases of brucellar and tuberculous ISI were excluded from the study. The following data was collected for each patient: (i) demographics (age at diagnosis, gender, and risk factors for septic arthritis); (ii) clinical characteristics (temperature using infrared tympanic thermometry, and site and intensity of pain at admission graded from 0–10 according to the visual analogue scale [VAS]); (iii) initial diagnosis; (iv) time to ISI diagnosis (time between the first clinical signs and confirmed diagnosis). Fever was defined as a temperature above 37.8°C.

Biological data (erythrocyte sedimentation rate [ESR] in mm/h, C-reactive protein [CRP] in mg/l [N < 5 mg/l]), leukocytes in g/l (leukocytosis defined as leukocyte number > 10 G/l), and microbiological data (mean isolation rate and type of pathogenic agent isolated) were also recorded.

MRI sequences including at least T1, T1 with Gadolinium infusion, and T2 sequences were performed. ISI was suspected when low signal intensity on T1 and high signal intensity on T2 were observed on the focused MRI slices.

The antibiotic therapy used as well as the clinical and biological evolution of ISI were analysed.

This study did not require formal ethical approval under French research regulations.

## Results

Overall, 39 patients were diagnosed with ISI between 1995 and 2011, comprising 16 men and 23 women (sex ratio M/F: 1/1.44) with a mean age of 39.7 ± 18.1 years [15.3-87].

Five cases occurred during the post-partum period, on average 9 days (range 1–30) after delivery, with the mean age of mothers being 29.8 ± 6.1 years. The involvement of the sacroiliac joint (SI) was always unilateral, with a left side predominance in 59% of cases (n = 23). At diagnosis, 17 out of 39 patients (44%) were febrile (mean temperature 37.8 ± 1°C).

The site of the pain was varied. Lumbogluteal pain was the most common (n = 36), although psoitis (n = 2) and hip pain (n = 1) were also reported. Manipulations of the SI joint were performed in seven cases (17.9%) and were always painful. Pain on admission, as evaluated in 16 patients, was intense (VAS = 7.3/10). Opioids were required for nine of the 23 patients without an available VAS score.

Only in five cases (12.8%) was the diagnosis of ISI suspected on admission. A variety of other diagnoses were suggested: sciatica (n = 15), spondylodiscitis (n = 4), common mechanical low back pain (n = 2), septic arthritis of the hip (n = 2), inflammatory sacroiliitis (n = 2), bacteremia not initially suspected to be osteoarticular in origin (n = 2), involvement of the psoas (haematoma n = 1 and abscess n = 1), multilocular sclerosis flare up (n = 1), intrasacral nodule (n = 1), sacral bone metastasis (n = 1), and sigmoiditis (n = 1). In one case, the initial diagnosis could not be identified precisely.

Time to diagnosis was evaluated with certainty in 36 cases, but remained long (mean 43.3 ± 69.1 days).

Regarding patient characteristics, seven patients were found to be immunosuppressed (corticosteroids n = 3, corticosteroids + azathioprine n = 1, neoplasia n = 2, and hypogammaglobulinaemia n = 1). In the days prior to admission, 11 patients reported a dental (n = 5), cutaneous (n = 3), or genitourinary (n = 3) infection. Two patients experienced trauma to the pelvis, while one was an intravenous drug user. Two patients reported intramuscular injections of non-steroidal anti-inflammatory drugs in the days preceding the onset of symptoms, while one patient was diabetic.

Biological inflammatory syndrome was variable. In all patients, CRP (n = 39) was elevated (mean level 149 ± 115.3 mg/l), with an increased erythrocyte sedimentation rate (ESR) (n = 27) (mean rate 71.1 ± 30.4 mm in the first hour). Increased levels of fibrinogen (n = 13) were found (mean level 6.3 ± 1.6 g/l), although leukocytosis (n = 32) was only observed in 46.8% of patients (mean leukocytes 10.4 ± 3.5 g/l).

A standard radiography of the frontal pelvis or focussed on the SI joint was performed in 33 patients at admission, with 39.4% of cases being normal (n = 13). In total, 27 abdominopelvic CT scans were carried out, revealing infectious arthritis of the SI in < 50% of cases (n = 13) and a psoas abscess in 29.6% (n = 8). Six of the CT scans were normal. Bone scans (n = 14) showed hyperfixation of the affected SI in 13 cases. In the patient exhibiting a normal bone scan, the examination was conducted on the second day following the onset of symptoms. MRI (n = 27) showed arthritis of the SI in 25 cases. In two cases where MRI was considered normal, the scan was carried out for suspected spondylodiscitis and thus, only explored the lumbar spine.

A pathogenic agent was isolated in 33 cases by means of articular puncture (n = 16), blood culture (n = 14), cytobacteriological examination of urine alone (n = 2), or puncture of the psoas (n = 1). In patients diagnosed on the basis of articular puncture, blood cultures were negative. Gram-positive cocci were the most frequently isolated pathogenic agents (n = 26), with a predominance of staphylococci (n = 21). *Pseudomonas aeruginosa* were isolated in three cases (Table 1). In the immunosuppressed patients, there was no predominant pathogenic agent (Table 2). All patients received antibiotic therapy (mean duration 3.01 ± 1.21 months, range 0.5-8). Antibiotic therapies consisted of rifampicin + oxacilline or rifampicin + ofloxacin for *staphylococci*, amoxicillin + gentamycin, or ceftriaxone + ofloxacine or cefotaxime + gentamycin or amoxicillin-clavulanic acid + ofloxacine when *streptococci* were involved. When *Pseudomonas aeruginosa* was isolated, tazocilline + ciprofloxacine or colistin + ceftazidime or ceftazidime + amikacin were used. An association of amoxicillin + rifampicine was used for *E*. *Coli* treatment.

**Table 1:** **Microorganisms isolated from the patients in our cohort**(**n** = **33**)

**Microorganism isolated**	**n**
Methicillin sensitive *Staphylococcus aureus*	16
Coagulase negative *Staphylococcus*	5
*Streptococcus*	5
*Pseudomonas aeruginosa*	3
*Escherichia coli*	1
*Propionibacterium acnes*	1
Polymicrobial	2

**Table 2:** **Microorganisms isolated from Immunosuppresed patients** (**n** = **7**)

**Micoorganism isolated**	
*Staphylococcus lugdunensis*	n = 1
Methicillin sensitive *Staphylococcus aureus*	n = 2
*Pseudomonas aeruginosa*	n = 1
*Propionibavterium acnes*	n = 1
Polymicrobial (Fusobacterium, Staphylococcus nominis, Streptococcus C)	n = 1
No pathogeneic agent	n = 1

Evolution was favourable in the majority of cases (n = 37), although one death occurred in a fragile patient, while another patient relapsed.

In 21 cases, patients complained of residual pain the SI region (n = 17), lumbar spine (n = 3), or radiculalgia (n = 1) during follow-up. In all cases, the pain was mechanical, while two patients reported limping.

**Figure 1:**
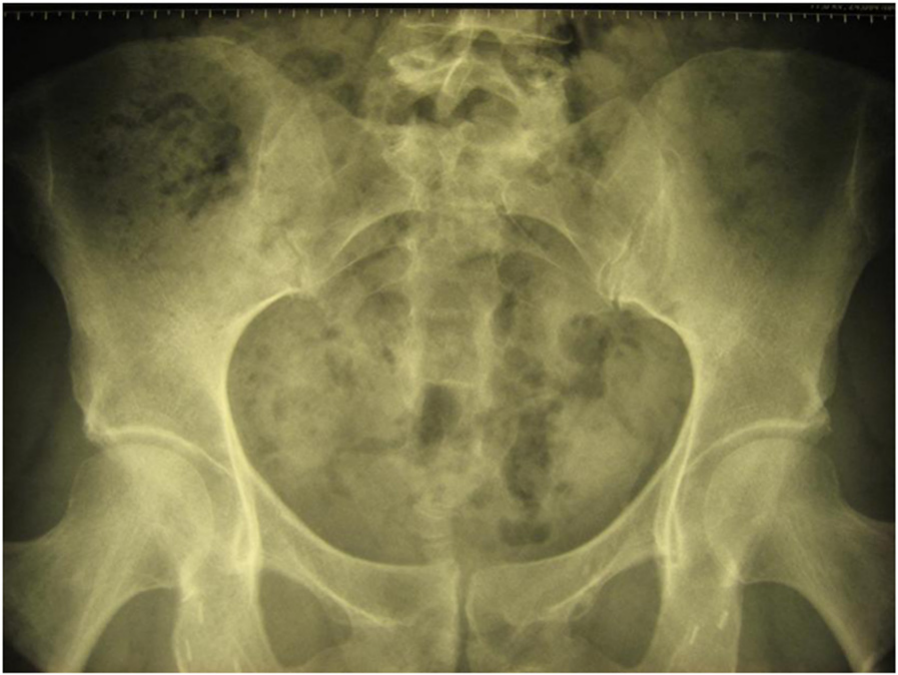
**Right**-**sided sacroiliitis persisting 1 month after diagnosis.**

**Figure 2:**
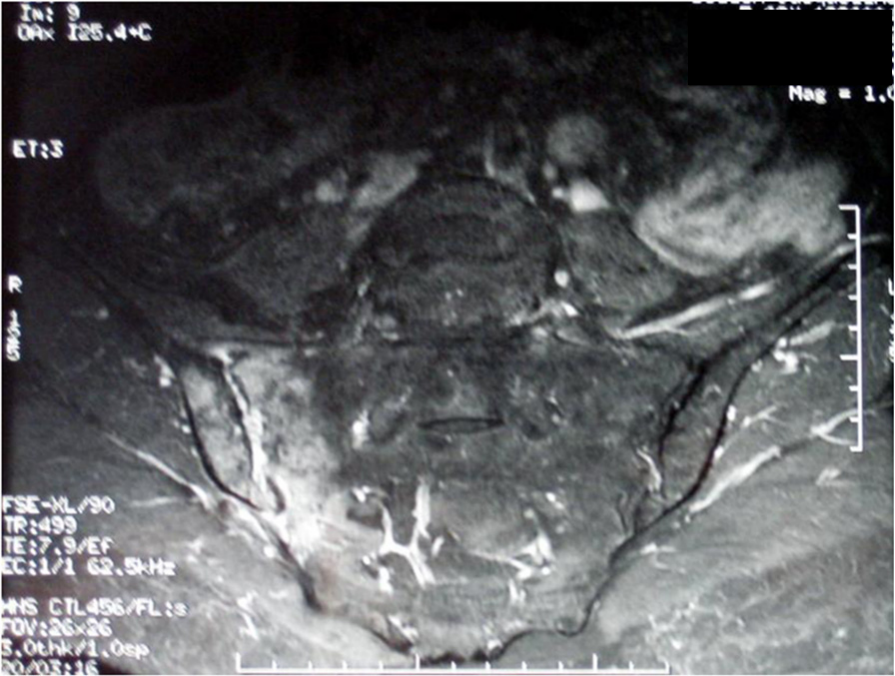
Magnetic imaging resonance scan showing persistent hypersignal of the right sacroiliac joint 3 months after diagnosis.

Surveillance by imaging was too heterogeneous to be accurately assessed. For 27 patients, follow-up involved radiography, CT scans, or MRI (Figure 1). The persistence of pathological signs was noted on MRI performed between 5 weeks and 19 months after diagnosis (n = 4) (Figure 2).

## Discussion

This report presents the largest documented series of ISI to date. Three previous studies only examined isolated cases in reviews [1-3]. The first review reported cases collected between 1878 and 1990, the second between 1990 and 1996, and the third between 1996 and 2009 [1-3]. In addition, we reviewed cases published since 2009 [4-13] (Table 3).

**Table 3:** Literature review of infectious sacroiliitis cases

	**Zimmerman**	**Mancarella**	**Literature view**	**Our cases**
	***et al***. [3]	***et al***. [1]	**2009**-**20011**	
			[4-13]	
No. of cases	177	97	36	39
Age (years), mean [range]	20.4	29.8	21.2	39.7 ± 18.1
	[0.75-72]	[2.5-80]		[15.3-87]
Sex ratio (M/F)	1/0.85	1/1.56	1/0.9	1/1.44
Pregnancy or per-partum	6 (3.4)	7 (7.2)	2 (3.9)	5 (12.8)
Fever				
Yes			7 (35.3)	16 (41)
NG	NG	NG	32 (62.7)	
Site of pain				
Side affected	Unilateral	NG	Left: 6(60)	Left: 23 (59)
	175 (98.9)			
Lumbogluteal	NG	NG	40(78.9)	36(92.3)
**Microorganisms**				
CG+	264 (81)	59 (61)	23 (63.9)	26 (78.8)
*Staphylococcus*				
MRSA	0(0)	0(0)	2 (3.9)	0(0)
MRSA	229 (86.7)	41 (69)	20 (86.9)	21 (80.8)
*Streptococcus*	29 (10.9)	17 (29)	3 (13.1)	5 (13.8)
BG				
*P*. *aeruginosa*	17 (31)	2 (17)	0 (0)	3 (75)
*Salmonella* sp	16 (29.1)	8 (67)	1 (50)	0
*E coli*	8 (14.5)	0 (0)	0 (0)	1 (25)

The prevalence of ISI is rare, involving between 1 and 2% of septic arthritis cases, which is probably due to the poor vascularisation of this joint, resulting in a low risk of infection via the haematogenous route [1,14].

ISI diagnosis is difficult owing to its clinical heterogeneity and the lack of symptom specificity [15], with some authors referring to it as a “diagnostic challenge” [3,16]. Similarly to our series, the most frequent clinical sign recorded in other reports was lumbogluteal pain [1-4,6,10-12], whereas coxofemoral pain, pubalgia, abdominal pain, and psoitis were also observed [1,3,9]. Although rarely performed, manipulation of the SI joint is often very painful [3,8,10-12].

The presence of fever is variable (41% of cases in our study *vs*. 35.3% in the literature). In an earlier review by Vyskocil, fever was found to be more common (75%) [2]. In pregnant women, fever may be absent in 33% of cases [16]. Thus, a diagnosis of ISI is rarely suspected on admission. As shown in our study, the clinical picture may be misinterpreted as sciatica or spondylodiscitis. Data reported in the literature does not allow for accurately estimating a diagnostic error ratio.

The heterogeneity of clinical signs associated with ISI may be accounted for by the anatomical structure of the SI [14]. The first and second sacral roots pass near the SI joints, with the joint capsule being bordered by the psoas muscle in front and the gluteal and pyriformis muscles behind. Depending on the capsular region involved in the arthritis, the pain may be gluteal or inguinal, mimicking damage to the hip. Doita [14] estimated that in 10% of cases, the involvement of the anterior joint capsule was responsible for the peritoneal irritation, which caused the painful abdominal symptoms sometimes described.

This atypical clinical presentation appeared to explain the lengthy time to diagnosis, which was rarely reported in the literature. In our study, the mean duration was 43.3 ±69.1 days (range 2–365) in contrast to a case series of ISI reported in pregnant women with a time to diagnosis ranging from 2 to 32 days [16].

Age-related changes to the SI, such as decreased vascularisation and mobility that limit the risk of bacterial colonisation, may explain the low frequency (<4%) of ISI in individuals aged >60 years [14] and its increased incidence in children [8]. The greater demands on the SI joint in pregnant women due to increased weight and hormone-induced changes to the pelvic conformation account for the higher frequency of ISI cases during pregnancy, in the immediate post-partum period, or following abortion (12.8% in our study and up to 7.2% in the literature) [7].

Generally, the infection is unilateral, with a preference for the left side (59% in our study and 60% in the literature, with the exception of a Taiwanese study reporting predominantly right-sided or bilateral infections [17]). During pregnancy, the infection appears to be bilateral more often (13.3% of cases) [16], although this was not observed in our study.

Risk factors, such as intravenous drug use, pelvic trauma, infectious endocarditis, haemoglobinopathy, immunosuppressive treatment, as well as cutaneous, respiratory or genito-urinary infections [1,3,16,18], are often found in ISI cases. In our study, the frequency was 30.9% compared with 44–55.5% reported in the literature [1,17].

Polynuclear neutrophil leukocytosis in addition to increased levels of CRP and ESR are standard features of ISI, while being inconsistent and non-specific [1,14,17].

MRI is the reference examination for establishing the diagnosis of ISI. This examination also enables clinicians to assess whether the infection has spread to the adjacent muscular structures, as observed in 48.1% of cases in our study [17-19]. In our trial, MRI directed the diagnosis towards ISI, provided that the slices were made through the SI. Consequently, MRI of the lumbar spine and SI should be systematically performed in a febrile patient with lumbogluteal pain, particularly in the case of pregnancy. There are no typical features differentiating spondylarthropathies from ISI. MRI signal anomalies persist for several months, even when clinical and biological improvement appears promising [20]. Furthermore, MRI is likely to play a role when the clinical evolution is unfavourable. As to other types of scans, although bone scans are not specific, they may be useful in localising the infection. However, bone scans may be misleading when carried out early, notably 3 days prior to the evolution of symptoms, as was observed in one of our cases [3,19]. Although CT scans provide an accurate assessment of the bone structure, they may confuse the diagnosis. In our study, CT scans were normal in 22.4% of cases when performed early [21], although they may be used while performing a biological procedure (*i*.*e*., biopsy, arthrocentesis, drainage). Standard radiography does not facilitate early diagnosis, as it is often normal [19]. Positron emission tomography with fluorine-18 fluorodeoxyglucose appears to be an interesting technique for the very early diagnosis of ISI, even before MRI or CT scans reveal any anomalies [9].

The frequency of ISI without any identified pathogenic agent has tended to decrease over time, from 27% in the literature review of Mancarella *et al*. [1] to 15.4% in our series. When a microorganism was identified, Gram-positive *Staphylococci* were the predominate pathogens, irrespective of the observation date. Since 2007, methicillin-resistant *Staphylococcus aureus* has emerged as a cause of ISI, including community-associated methicillin-resistant *Staphylococcus aureus*[8,17,22].

When the infection was caused by a Gram-negative bacillus, two microorganisms were frequently encountered, namely, *Salmonella* spp. and *P*. *aeruginosa*. The latter has increased in frequency since 1990. *P*. *aeruginosa* is the most commonly encountered Gram-negative bacillus in immunosuppressed patients, hospitalised patients, and intravenous drug users [1], while *Serratia marcescens* is the second most common Gram-negative bacillus in intravenous drug users [23]. *Salmonella* spp. is more frequent in children than adults [22]. *Escherichia coli* was found in only 2.4% of cases, usually in the context of a known urinary tract infection [3].

Clinical and anamnestic factors may guide the bacteriological investigation. *Streptococcus* is more frequent agent when functional gynaecological signs are present. In contrast, diarrhoea or digestive problems are not systematically found when *Salmonella* is the causative agent [3]. In addition to pyogenic microorganisms, *Brucella* is a frequent cause of septic arthritis in certain parts of the world, such as Turkey [24]. A study of 202 brucellar arthritis cases reported the involvement of the SI joint in 60.6% of cases. Furthermore, leprosy may also affect the SI, mimicking spondylarthropathy [25].

The definitive microbiological diagnosis may be based on blood cultures, joint fluid by CT-guided percutaneous puncture, or surgical investigations [3,15]. When performed, blood cultures are positive in 57.6% [17] to 69% [1] of adults and 45.5% of children [17]. Blood cultures contributed less (42.4%) in our study compared with the literature.

The duration of antibiotic therapy was extremely variable in our study (2–34 weeks) compared with the literature (up to 46 weeks) [26]. No consensus exists as to the duration of antibiotic therapy in ISI, but it seems reasonable to propose parenteral treatment for 2 weeks followed by oral treatment for 6 weeks in the case of ISI caused by pyogenic bacteria, which is in accordance with the SPILF (*Société de Pathologie Infectieuse de Langue Française*) recommendations for the treatment of infectious spondylodiscitis [27]. There appears to be no justification in prolonging treatment beyond 6 weeks. British recommendations for the treatment of infectious spondylodiscitis favour parenteral treatment for 3 weeks followed by oral treatment for a total of 6–12 weeks [28]. However, antibiotic therapy for more than 6 weeks does not reduce the risk of relapse [29].

In the absence of any identified microorganism, it is preferable to consider antibiotic therapy active against *Staphylococcus*[14], which in the case of failure, should be extended to include Gram-negative bacilli [17], in line with the SPILF recommendations for spondylodiscitis [27].

While exceptional, surgical treatment may be proposed in the case of abscesses, proven osteomyelitis, bone involvement or necrosis, and failed antibiotic therapy [1]. Short-term evolution is usually favourable, with a single death being observed in our study and none in the recent literature. Long-term lumbogluteal pain intensifying during daytime activities was reported to persist in more than one-third of cases in the literature and in a greater proportion of cases in our study (33% *vs*. 43.5%) [1,16,17]. Drug addicts, however, have a higher risk of abscesses, osteomyelitis, and relapse after stopping antibiotic therapy [23]. In the event of ISI occurring during pregnancy, appropriate antibiotic therapy should be initiated. Peridural analgesia is contraindicated, and delivery should be by caesarean section in order to avoid escalating joint pain during labour [3,4].

As in all retrospective studies, our clinical and paraclinical data is not exhaustive, although the multicentre nature of our study limited any bias in recruitment.

## Conclusion

In conclusion, the diagnosis of ISI is long and difficult to establish. However, MRI of the lumbar spine and SI in a febrile patient with gluteal pain should allow for a more rapid diagnosis of ISI. This diagnosis should be suspected in patients with confusing clinical symptoms (coxofemoral pain, pubalgia, abdominal pain, and psoitis), and MRI of the SI or bone scintigraphy should be performed depending on the rate at which symptoms progress. Short-term evolution is largely favourable, although consensus on the duration of antibiotic therapy and the modalities of patient follow-up remain to be established.

## Competing interests

The authors declare no financial support, grants, or funding for this work. None of the contributing authors have any conflicts of interest, including specific financial interests, relationships, and affiliations relevant to the subject matter discussed in this manuscript.

## Authors’ contributions

All authors were involved in drafting the article or revising it critically for important intellectual content, and all authors approved the final version to be published. Study conception: MH, MS Acquisition of data: MH.

## Pre-publication history

The pre-publication history for this paper can be accessed here:

http://www.biomedcentral.com/1471-2334/12/305/prepub
